# The age at first consumption of forage in calves and its effect on growth and rumination in the short- and long-term

**DOI:** 10.1186/s40104-023-00885-6

**Published:** 2023-07-24

**Authors:** Jianxin Xiao, Tianyu Chen, Rong Peng, Gibson Maswayi Alugongo, Hui Yang, Muhammad Zahoor Khan, Shuai Liu, Yulin Ma, Jingjun Wang, Wei Wang, Yajing Wang, Shengli Li, Zhijun Cao

**Affiliations:** 1 Key Laboratory of Low Carbon Culture and Safety Production in Cattle in Sichuan, Animal Nutrition Institute, Sichuan Agricultural University, Chengdu, 611130 People’s Republic of China; 2 State Key Laboratory of Animal Nutrition, International Calf and Heifer Organization, College of Animal Science and Technology, China Agricultural University, Beijing, 100193 People’s Republic of China

**Keywords:** Age at first forage consumption, Dairy calf, Growth, Nutrient digestibility, Rumen fermentation, Rumination

## Abstract

**Background:**

Previous investigations into the effect of dietary forage on calf performance have been inconsistent, and there is a paucity of information exploring the effect of age on the growth performance and rumination of calves. Eighty-four female Holstein calves (41.5 ± 4.2 kg) were enrolled at birth, a subset of the calves were fed calf starter only (CON, *n* = 21) while the rest (*n* = 63) were classified into three treatment groups: the early (EHAY, *n* = 26, 5.1 ± 0.8 d), the middle (MHAY, *n* = 21, 7.9 ± 0.8 d) and the late (LHAY, *n* = 16, 12.1 ± 1.4 d) hay consumers. The short-term effect of the age at first forage consumption (AFF) on calves’ feed intake was monitored until d 84. In addition, the long-term effects of AFF on body weight, structural growth and rumination behavior were recorded until d 196. Rumen samples were collected on d 1, 7, 35, 84 and 196 to analyze the rumen fermentation, while fecal samples were collected from d 78 to 84 to estimate digestibility parameters.

**Results:**

Treatment had no effect on feed intake. While, the EHAY calves tended to have lower BW and ADG compared to LHAY and CON calves. Several total-tract apparent digestibility parameters and digestible nutrients intake were significantly lower in EHAY calves compared with CON and LHAY calves. Calves in the EHAY group tended to begin ruminating ealier, while CON calves were the latest (12.3 vs. 15.5 days of age). A treatment and time interaction was present for rumination time due to greater rumination in calves consuming hay compared to CON calves in week 10 to 12, the differences in rumination disappeared afterwards, no long-lasting significant differences in the rumination and rumen fermentation parameters were found between treatments.

**Conclusions:**

In conclusion, this study showed that hay consumption earlier in life (in the first week, around 5 days of life) could negatively affect the growth of the calf in the short and long term. Compared to consuming hay from the second week (around 12 days of life) or feeding concentrate only without hay, starting to consume hay from the first week could compromise nutrient digestibility and digestible nutrient intake independent of developing rumination behaviour and rumen fermentation.

## Introduction

In the last one hundred years, forage feeding to preweaned calves has remained a key concern in calf nutrition. Before the 1950s, forage feeding was generally encouraged in preweaned calves, as it was believed to improve rumen development [1, 2]. In the 1960s, research emerged challenging the fact that forage feeding improved rumen development to the same degree as calf starter concentrate [3]. Starter concentrates are rich in rapidly fermentable carbohydrates, and when consumed alone or in higher proportions of the diet, they produce greater amounts of volatile fatty acids (VFA), especially butyrate and propionate [4]. On the other hand, dietary forage inclusion results in a higher proportion of acetate [5], which does not stimulate rumen papillae growth to the same extent as butyrate and propionate [6, 7]. Furthermore, when fed together, forage might increase gut fill due to its low fiber digestibility and ruminal fermentation rate compared to starch and sugars in concentrates, thus curbing the energy density available to the calf [2, 8, 9]. Therefore, in lieu of concerns regarding rumen development, many farms do not offer forage to calves during the preweaning period to promote grain intake [10]. Moreover, feeding forage early in life might increase labor demands and the wastage of high-quality forage.

In the 2000s and 2010s, interest in research on feeding forage to preweaned calves broadened to other areas such as type, amount, and particle size of forage supplemented in the diet. Although results have been inconsistent, several studies have shown that providing small amounts of high quality forages, such as alfalfa and oat hay, might improve the rumen fermentation environment and development, while enhancing calf performance [11, 12]. Forage encourages chewing and rumination, two important activities that promote saliva production and rumen buffering [13–15]. Additionally, forage could reduce rumen plaque formation [16, 17] and subsequently increase the absorptive surface area of the rumen epithelium, alleviate VFA accumulation and maintain appropriate rumen fluid pH [18–20]. Additionally, providing forage was associated with less non-nutritive oral behavior in calves [21], suggesting that providing forage early in life is essential for calf welfare and development. Besides, the anatomical development of the rumen depends on the physical form of the diet [22, 23]. In this regard, calves supplemented with forage in their diet have reported greater rumen volume [7, 17], weight [24], wall thickness and papillae [25], partially explaining the improved growth and performance in calves.

Although research indicates that feeding forage preweaning calves can stimulate rumen development, the most appropriate age at which the forage should be introduced remains unresolved. In a limited number of studies, the greatest DMI and growth performance have been observed in calves offered forage (alfalfa or oat hay) from the second rather than the fourth or fifth week of age [26–28]. However, it is unknown whether feeding forage at a much younger age (such as right after birth) would be more effective in supporting growth. Due to its pseudo-monogastric nature, calves have an undeveloped rumen at 2 weeks of life [5] and cannot utilize forages. Interestingly, it was recently observed that even without providing forages, newborn calves began to eat straw bedding as early as 3 days of age [29]. The researchers further showed that the age at which calves began to eat the bedding material was moderately positively correlated with the age at first rumination (*r* = 0.55) [29]. Since rumination behavior is a key biological marker of rumen development, the occurrence of rumination distinguishes ruminants from monogastric animals [30]. Thus, these results imply that eating forages at the earliest possible age might improve rumen development and further benefit the growth performance of calves. All the previous studies investigated the optimal time of feeding forage based on the age at which forage was provided or available, rather than when calves voluntarily started to consume the forage by themselves. Thus, in this study, we focused on the actual age calves began to consume forage voluntarily and its effects on calf performance. This study provides insights into the appropriate time preweaned calves should consume forage for optimal calf feeding and management. We hypothesized that divergence in age at first forage consumption (AFF) could result in differences in rumination and growth performance among calves, as observed in the development of the rumination, feed intake, nutrient digestibility and growth performance.

## Materials and methods

### Animals, treatments and feeding

This study was conducted at the Modern Farming Co., Ltd. (Baoji, Shanxi, P. R. China) from November 2020 to April 2021. The experiment and animal procedures were done according to the Guidelines for Care and Use of Laboratory Animals of China Agricultural University (Beijing, China) and approved by the Animal Ethics Committee of China Agricultural University (Approval No. AW82211202-1-1).

Eighty-four newborn female Holstein calves (41.5 ± 4.2 kg of initial BW) were enrolled in this study. If an experimental calf died unexpectedly before 84 days of age, it was replaced with a new one immediately and data from the dead calf discarded. Calves with a normal birth weight (≥ 30 kg) were separated from their dams and then transferred to individual hutches before receiving 4 L of colostrum (> 22% Brix) within 1 h of life. As an indicator of health, serum total protein (≥ 5.5 g/dL) was measured 24 h after colostrum feeding to determine the passive transfer of immunity. Calves were offered pasteurized whole milk twice daily at 0800 and 1500 h from d 2 to 56, as follows: 8 L/d from d 2 to 21, 10 L/d from d 22 to 42, 8 L/d from d 43 to 49, and 6 L/d from d 50 to 52, 4 L/d from d 53 to 55 and 2 L/d on d 56, afterwards calves were weaned completely off milk on d 57. Milk was pasteurized using a milk pasteurizer, where milk temperature was elevated and held at 60 °C for 30 min and then cooled to 37 °C. All calves were raised in the hutches with a fenced area from d 1 to 84. The inside dimensions of the fenced area were approximately 1.15 m × 1.8 m, and the outside dimensions were approximately 1.2 m × 1.6 m. The hutches were cleaned and sterilized before introducing calves, according to the standard operating procedures of this farm. Sawdust was used as bedding material and was renewed weekly. Two buckets containing solid feed (for starter concentrate and oat hay) were hung inside the hutch, and another two for water and milk were attached on the outside and in front of the hutch. All calves had free access to solid feed and water. From d 85 of life, calves were transferred to a barn and housed in one big group feeding the same diet.

### The age at first forage consumption

The age at first forage consumption was defined as the first day a calf ingested forage (> 0 g based on the amount of oat hay offered minus the amount refused). The bucket could hold around 400 g of oat hay once every time it was fully loaded. To avoid wasting forage when calves were feeding, we provided only 50 g daily until calves began to eat the oat hay. Besides, we did not observe any indication of forage wastage inside the calf pens during the experimental period. Thus we confirm that forage was consumed by calves rather than wasted and that the age of forage consumption was calculated accurately in the current study.

### Experimental design and diets

From d 4, all calves were randomly assigned to two feed exposure treatments, which included: 21 calves that were offered milk and calf starter concentrate only (CON) from d 4 to 84 and 63 calves that were offered milk, calf starter concentrate and oat hay (HAY) from d 4 to 84. Complete pellet starter concentrate and oat hay were offered free choice in individual buckets every morning for ad libitum intake.

Among the HAY calves, we retrospectively assigned them into 3 groups depending on when they started to consume hay. The three groups were: early (EHAY, *n* = 26), middle (MHAY, *n* = 21) and late (LHAY, *n* = 16) hay consumers. The ages at which the calves began to consume forage were d 4 to 6 for EHAY, d 7 to 9 for MHAY, and d 10 to 15 for LHAY. From d 85 to 133, all calves were offered the same starter concentrate (90% of diet) top-dressed with oat hay (10% of diet) ration (TDR, concentrate spread over hay), allowing the calves free access to either concentrate or hay. Calves were then offered a novel total mixed ration (TMR) from d 134 to 196. The TMR was fed once daily at 1000 h immediately after disposal of the orts. The TMR composition included alfalfa hay (39.4%), corn silage (32.5%), soybean meal (13.5%), steam-flaked corn (11.7%) and premix compound (2.9%) on a DM basis.

### Sample collection

#### Feed sampling and analysis

Daily feed intake was recorded based on the amount offered and refused by each calf from d 4 to 84. The age at first forage or concentrate consumption was recorded. Forage feed intake ratio (FIR) was calculated based on hay intake divided by total solid feed intake (including concentrate and hay, DM basis) from d 4 to 84. The DMI and FIR data were calculated and analyzed for the individual calf to determine the feed preference before and after weaning. Both types of feed were offered in sufficient amounts to ensure at least 10% orts. Representative samples of calf starter concentrate, oat hay and milk were collected weekly and immediately frozen at −20 °C until further analyzed for dry matter (DM, method 930.15 of the Association of Official Analytical Chemists (AOAC)), crude protein (CP, method 988.05 of AOAC), ether extract (EE, method 920.39 of AOAC) and crude ash (Ash, method 924.05 of AOAC) following AOAC methods [31] for starter and oat hay samples. Starch content was analyzed using a total starch assay kit (Megazyme, Bray, Ireland) based on AOAC method 996.11 [31]. Neutral detergent fiber (NDF) and acid detergent fiber (ADF) were analyzed using the ANKOM fiber analyzer (A2000i; American ANKOM, Macedon, NY, USA), as described by van Soest et al. [32]. The particle size of the oat hay and TMR were analyzed using a 3-sieve (19, 8, and 4 mm) Penn State Particle Separator. The nutritional composition and particle size distribution of all the diets are listed in Table 1.

**Table 1:** Chemical composition and particle size distribution in the starter, hay, milk and TMR

Item	Calf starter^1^	Oat hay^2^	Milk	TMR^3^
Chemical composition				
Dry matter, %	88.7	88.0	13.0	50.7
Crude protein, % of DM	22.8	6.4	27.4	17.4
Fat, % DM	-	-	30.8	-
SNF^4^, % DM	-	-	69.2	-
Starch, % DM	32.3	0.6	-	17.5
NDF, % of DM	17.0	47.7	-	34.6
ADF, % of DM	10.5	25.6	-	25.6
ASH, % of DM	7.4	4.6	-	-
EE, % of DM	1.7	1.4	-	3.0
ME^5^, Mcal/kg of DM	2.9	2.2	5.4	2.5
Particles^6^, %				
Long	-	39.0	-	8.4
Medium	-	25.1	-	42.0
Short	-	18.1	-	14.2
Fine	-	16.4	-	35.4

^1^Calf starter was supplied by Modern Farming Co., Ltd., containing 33.6% corn, 18.8% fermented soybean meal, 14.1% soy hulls, 10.5% soybean meal, 6.3% wheat bran, 6.3% wheat flour, 5.4% whey powder, 5% premix compound (contained vitamin A 200,000 IU/kg, vitamin D 25,000 IU/kg, vitamin E 2,000 IU/kg, manganese 0.6 g/kg, iron 0.4 g/kg, copper 0.5 g/kg, zinc 2 g/kg, cobalt 4 mg/kg, iodine 16 mg/kg, and selenium 4 mg/kg) on a dry matter (DM) basis

^2^Oat hay was cut less than 2.5 cm using a stationary mixer (20 m^3^, Trioliet Co., Ltd., Holland)

^3^The total mixed ration (TMR) was composed of 39.4% alfalfa hay, 32.5% corn silage, 13.5% soybean meal, 11.7% steam flaked corn and 2.9% premix compound (contained vitamin A 300,000 IU/kg, vitamin D 80,000 IU/kg, vitamin E 2,000 IU/kg, Mn 2.0 g/kg, Cu 0.7 g/kg, Zn 2.5 g /kg, Co 20 mg/kg, iodine 15 mg/kg, and selenium 14 mg/kg) on a dry matter basis

^4^SNF: Solid non-fat

^5^ME for calf starter, hay, milk and TMR was calculated according to NRC (2001) [10] equations

^6^Samples for particle size were analyzed using a 4-sieve (19, 8, 4 mm and bottom) Penn State Particle Separator

#### Body weight and structural measurements

All calves were weighed on a digital scale every two weeks for the first 12 weeks (d 1, 14, 28, 42, 56, 70 and 84) and once in week 28 (d 196). Average daily body weight gain (ADG) and feed efficiency (FE, kilogram of DMI per kilogram of BW gain) per calf were calculated every two weeks up to 84 d. Withers height (vertical distance from the highest point of calf withers to the ground) and heart girth (the horizontal circumference of the calf body at the posterior edge of the scapula) were also recorded concurrently with BW.

#### Rumen and blood samples

Rumen fluid was collected on d 1, 35, 84 and 196 of age by a flexible esophageal tube (2-mm of wall thickness and 6-mm of internal diameter; SciTech Co., Ltd, Wuhan, Hubei, PRC) from all calves 2 h after the morning feeding. The first 10 mL of rumen fluid was discarded to avoid saliva contamination. Rumen fluid was filtered through 4 layers of cheesecloth. Rumen pH was measured immediately with a glass electrode pH meter (HORIBA Advanced Techno Co., Ltd., Osaka, Japan). 15 mL of the rumen fluid was preserved at −20 °C for later analysis of VFA and NH_3_-N concentration using gas chromatography [33] and the phenol-sodium hypochlorite colorimetric [34] methods, respectively.

Blood was sampled before the morning feeding on d 84 to determine the energy metabolism-related metabolites [(glucose, urea, beta-hydroxybutyric acid (BHB) and nonesterified fatty acids (NEFA)]. Briefly, blood was collected via the jugular vein using evacuated tubes containing no anticoagulant for serum separation. Blood was then centrifuged at 3,500 × *g* for 15 min in a centrifuge at 4 °C for separation of serum and then partitioned into aliquots and stored in 1.5-mL microcentrifuge tubes at −20 °C for later analysis. The concentrations of blood glucose, urea and BHB were measured by an automatic biochemical analyzer (Hitachi 7600, Hitachi High-Technologies Corporation, Tokyo, Japan) in accordance with the manufacturer's protocols. The level of NEFA was analyzed by calorimetry using commercial kits (A042-2–1, Nanjing Jiancheng Bioengineering Institute, China) per the manufacturer’s instructions.

#### Rumination behavior

In the current study, the rumination behavior was recorded for all calves using an ear-attached accelerometer (Monitoring Ear Tag Flex, Allflex® Livestock Intelligence eSense™; SCR Engineers Ltd., Netanya, Israel). Previous works have evaluated the feasibility of using acceleration sensors to monitor the rumination behavior in calves [35–37]. These studies reported the sensors as reliable tools for identifying and detecting rumination behavior. Briefly, rumination was recorded through an ear movement via a 3-dimensional accelerometer placed in the ear tag. The sensor was positioned on an electronic identification tag in the middle of the calf’s left ear. To minimize the risk of the sensor position influencing the recordings, all sensors were placed 6 cm from the concha and centered between the 2 typical major veins across the calves’ ear. Data from the sensors were collected through a router and sent to the local computer. The data were stored and processed in minutes by the Young Stock application and Heatime® Pro^+^ software (Allflex® Livestock Intelligence™; SCR Engineers Ltd., Netanya, Israel) on an hourly and daily basis per calf. Daily rumination time (min/d) was continuously recorded from d 1 to 196 and downloaded from the software for further analysis.

#### Total-tract apparent nutrient digestibility

After weaning, total-tract apparent digestibility was determined in all calves following the method used by Soltani et al. [38]. Twenty-one fecal grab samples (thrice daily at week 12, from d 78 to 84) per calf were collected directly from the rectum (at 0600, 1400 and 2200 h to account for 8-h intervals) to estimate apparent total tract nutrient digestibility by quantifying acid insoluble ash (AIA) as an internal marker in the feed (corrected for refusals) and fecal samples as described by van Kuelen and Young [39]. A calf was rectally stimulated with a sterile glove to facilitate the collection of the fecal samples. Daily individual calf fecal samples were composited (equal amount on a wet weight basis) into one sample per calf and frozen at −20 °C. The samples were further pooled by calf over a 7-day period to obtain a single composite (combined on an equal wet weight basis) until further analysis. Composite fecal samples were thawed at room temperature and placed on aluminium trays in an oven at 60 °C until completely dried (approximately 72 h). Dried fecal samples and representative feed samples (stater or oat hay) over the 7 d were ground through a 1-mm screen in a Wiley mill (Arthur H. Thomas, Philadelphia, PA, USA) and analyzed for their chemical compositions (CP, Starch, EE, Ash, NDF and ADF). Individual calf AIA and percentage of feed nutrient consumed were corrected based on the starter and oat hay intake over the 7 d for each calf. The equation used to calculate digestibility was as follows:

$$\mathrm{Nutrient\ digestibility,}\ \mathrm{\%}=100-\frac{100\ \times\ \left(\mathrm{nutrient\ in\ feces},\ \mathrm{ \% }\ \times \mathrm{ AIA\ in\ DM\ consumed},\ \mathrm{\%}\right)}{\mathrm{AIA\ in\ feces},\ \mathrm{ \% }\ \times \mathrm{ nutrient\ in\ consumed\ DM},\ \mathrm{ \%}}$$

The digestible nutrient intake was calculated based on the feed intake (g/DM) at 12 weeks of age (from d 78 to 84), the nutrient content of the feed (%, DM) and the coefficient of apparent digestibility of nutrients during that time. The equation used to calculate digestible nutrient intake was as follows:

$$\mathrm{Digestible\,nutrient\,intake}=\mathrm{nutrient \,content \,intake},\ \mathrm{g}\ \times\ \mathrm{ apparent \,digestibility \,of \,nutrient },\ \mathrm{\%}$$

$$\text{Nutrient content intake }=\left(\text{starter intake},\mathrm{ g }\times \text{ nutrient content of starter},\mathrm{\%}\right)+ (\text{forage intake},\mathrm{ g }\times \text{ nutrient content of forage},\mathrm{\%})$$

### Statistical analyses

A priori statistical power analysis using the primary response variable ADG was done to determine an adequate number of animals per treatment. We calculated the statistical power using G*Power software (version 3.1) to obtain a power (1 − β) of 0.80 and a type-I error probability (α) of 0.05. The effect size was determined from the previous results [28] and was 0.38 for ADG. From our calculation, a minimum of 80 calves were required to meet power requirements. Besides, the mortality rate during the preweaned period was estimated at 3%. To avoid missing data due to the death of a calf, four extra calves were included in the experiment. Thus, a total of 84 calves were used in the current study.

All raw data was summarized in the EXCEL sheet. All the data were checked for normality using the D'Agostino-Pearson omnibus normality test of GraphPad Prism version 8 (GraphPad Software Inc.) before analysis. The calf was the experimental unit. Data for milk, solid feed and nutrient composition intakes and FIR were summarized for each calf by week and analyzed for the calf period (week 1 to 12). Since the calves in CON group were not fed hay before d 84, data for hay DMI and FIR were analyzed for the HAY groups only. Data for BW, ADG and structural growth at d 1, 28, 56, 70, 84 and 196, and data for ruminal pH, NH_3_-N, and VFA at d 1, 35, 84 and 196 were analyzed over the overall period (d 1 to 196). As we did not record the DMI after d 84, data for FE were analyzed during the calf period. As data for daily rumination time was continuously recorded until the end of the experiment (d 196), it was summarized for each calf by week and analyzed over the overall period (week 1 to 28). All data were analyzed as a randomized complete block design using the MIXED procedure of SAS (version 9.4, SAS Institute Inc., Cary, NC, USA) with time (week or day) as a repeated measure. The model included the fixed effects of treatment, time and their interactions (treatment × time), and the random effects of calf within the treatment. Various covariance structures, including CS, Simple, UN, AR (1) and ANTE (1), were examined to find the best-fitted structure for the model based on the lowest Akaike information criterion. The initial BW was included as a covariate in the statistical analysis of BW and ADG. The initial structural growth measurements were included as covariates in the statistical analysis for structural growth performance. For all the analyses, significant differences were declared at *P* ≤ 0.05, with trends indicated at 0.05 < *P* ≤ 0.10.

## Results and discussion

### Distribution of age at first forage consumption

**Fig. 1:**
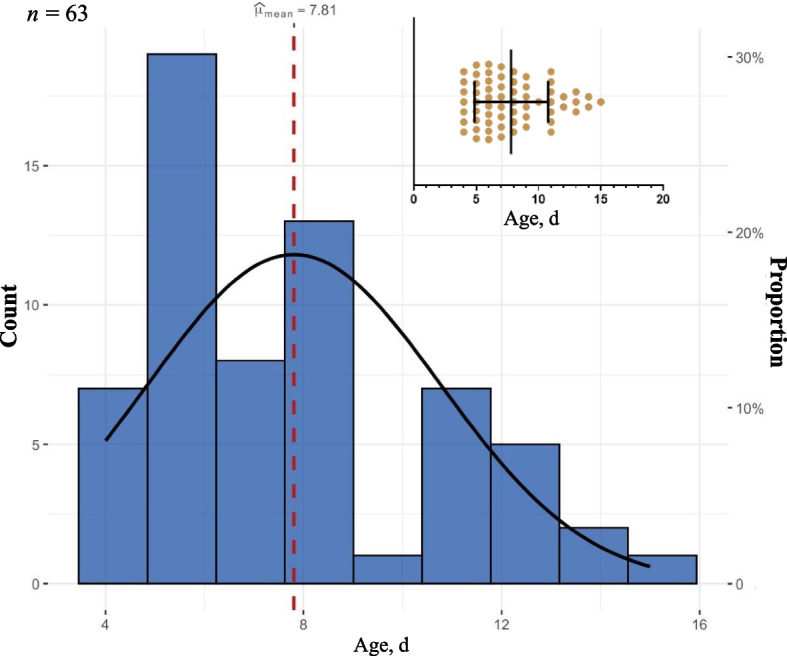
Distribution of calves based on the age at first forage consumption

The age at first forage consumption was defined as the first day a calf ingested forage. The AFF data were normally distributed, with the earliest AFF recorded at 4 d (the age of offering the forages) and the latest at 15 days of age. The range, average and standard deviation (SD) of AFF were 11, 7.81 and 2.95 d, respectively (Table 2 and Fig. 1), indicating that individual differences were relatively large. Based on these results, approximately 41.3% of the calves that began to consume forage at the earliest time possible were defined as the early hay consumers (EHAY, from d 4 to 6, 26 calves), the middle 33.3% as the middle hay consumers (MHAY, from d 7 to 9, 21 calves) and the remaining 25.4% as the late hay consumers (LHAY, from d 10 to 15, 16 calves).

**Table 2:** Descriptive statistics for the age at first forage and starter concentrate consumption and rumination behavior in the calves

Item	Number	Minimum	Median	Maximum	Mean	Max – Min	SD
AFF	63	4	7	15	7.81	11	2.95
AFC	84	4	6	12	6.54	6	2.22
AFR	84	6	13	27	14.4	21	4.71

*AFF* Age at first forage consumption, *AFC* Age at first starter concentrate consumption, *AFR* Age at first rumination behavior

### Feed intake and growth performance

**Fig. 2:**
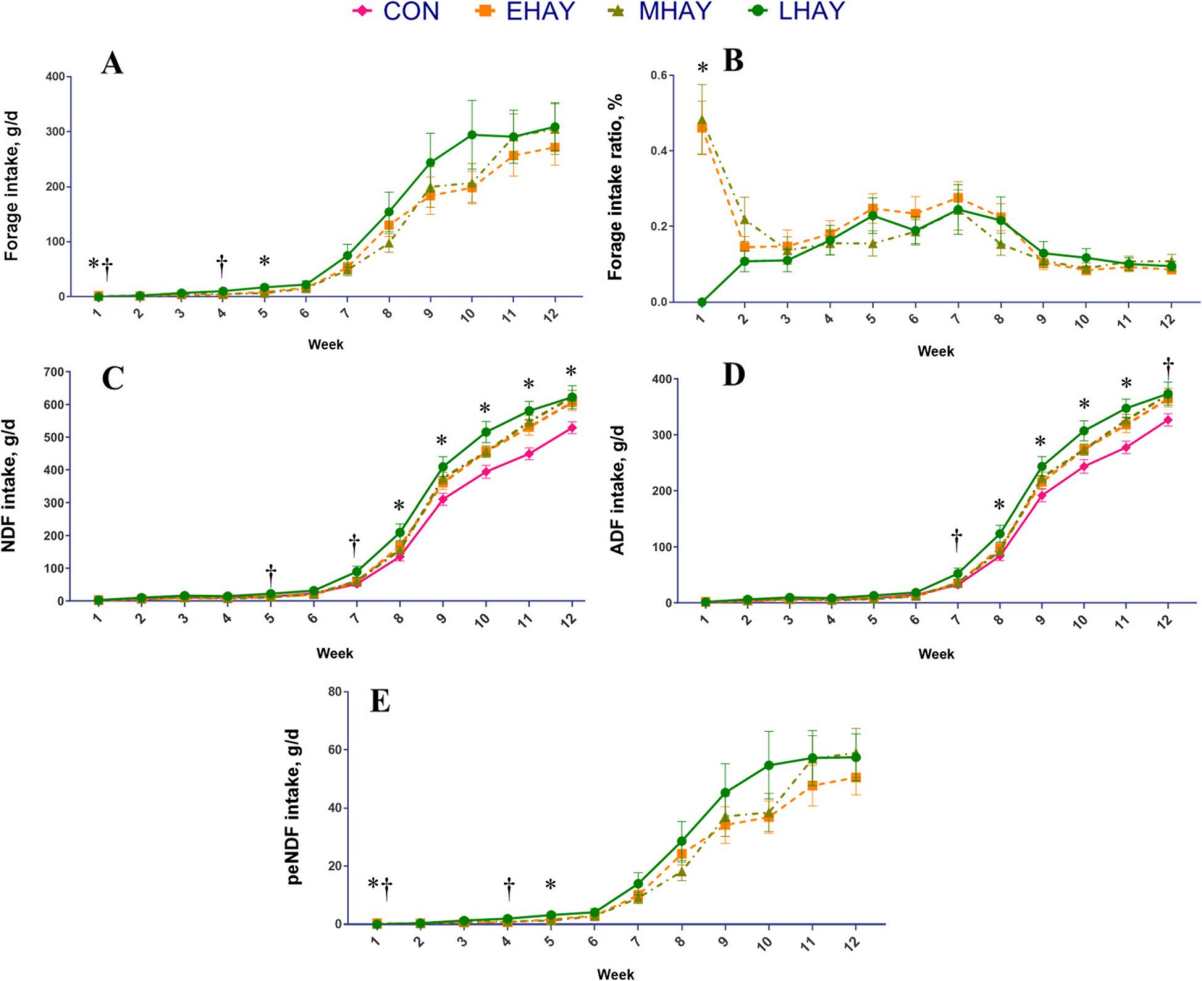
Weekly intakes of calves began to consume forages at different ages (CON, EHAY, MHAY and LHAY). **A** Weekly forage intake of dairy calves. Asterisks (*) indicate differences (*P* ≤ 0.05) between groups (week1: LHAY < EHAY and MHAY; week 5: EHAY and MHAY < LHAY). Daggers (†) indicate trends (0.05 ≤ *P* ≤ 0.1) between groups (weeks 1: MHAY < EHAY; weeks 4: EHAY < LHAY). **B** Weekly forage intake ratio of dairy calves. Asterisks (*) indicate differences (*P* ≤ 0.05) between groups (week 1: LHAY < EHAY and MHAY). **C** Weekly NDF intake of dairy calves. Asterisks (*) indicate differences (*P* ≤ 0.05) between groups (week 8: CON and MHAY < LHAY; week 9 and 10: CON < LHAY; week 11 and 12: CON < EHAY, MHAY and LHAY). Daggers indicate trends (0.05 ≤ *P* ≤ 0.1) between groups (weeks 5 and 7: CON, EHAY and MHAY < LHAY). **D** Weekly ADF intake of dairy calves. Asterisks (*) indicate differences (*P* ≤ 0.05) between groups (weeks 8, 9 and 10: CON < LHAY; week 11: CON < MHAY and LHAY). Daggers indicate trends (0.05 ≤ *P* ≤ 0.1) between groups (weeks 7 and 12: CON < LHAY). Error bars represent ± SE. **E** Weekly peNDF intake of dairy calves. Asterisks (*) indicate differences (*P* ≤ 0.05) between groups (week1: LHAY < EHAY and MHAY; week 5: EHAY and MHAY < LHAY). Daggers (†) indicate trends (0.05 ≤ *P* ≤ 0.1) between groups (weeks 1: MHAY < EHAY; weeks 4: EHAY < LHAY). CON = Calves were fed starter concentrate without forage from d 1 to 84, HAY = Starter concentrate and forage were fed to calves as a free choice way from d 1 to 84, EHAY = calves began to consume forage at the earliest (from d 4 to 6), MHAY = calves began to consume the forage from d 7 to 9, LHAY = calves began to consume forage at latest (from d 10 to 15)

The least square means for feed intake parameters are shown in Table 3. No differences were found in the starter, hay, total solid feed, total DMI and the ratio of hay to total solid feed intake between treatments. Treatment by time interactions were found for hay DMI intake and the ratio of hay to total solid feed intake among the three HAY groups (Table 3 and Fig. 2), such that EHAY and MHAY calves have a higher hay intake than the LHAY calves in the first week of life, while LHAY, which started to consume hay in the second week, tended to have greater intake than EHAY in week 4 and had greater intake than EHAY and MHAY in week 5. Similar hay intakes were observed during the experimental period for the rest of the weeks. Besides, a significant time effect was found for all the feed intake parameters (*P* < 0.01), due to dynamic changes in the feed intake parameters. The starter, total solid, and total DMI gradually increased in all groups, while the hay to total solid ratio decreased over time. The average hay to solid intake ratio in HAY groups was recorded as 31.3% in the first week of life, but rapidly decreased to 15.8% in the second week (*P* < 0.01, data not shown), suggesting that calves had the awareness to select the feed they would prefer to consume early life. After milk withdrawal, the ratio of hay to solid feed intake decreased significantly from 19.8% in week 8 to 11.5% in week 9 (*P* = 0.02, data not shown). Our previous results also showed that the ratio of hay to solid feed intake decreased over time, with calves preferring to consume more concentrate after weaning [40]. Given that dairy cows have a strong preference for sweet flavors that reflect higher energy levels [41], it is easy to understand why calves tend to increase the proportion of concentrate intake gradually over time. When the high-nutrient milk diet is withdrawn, and as they grow older, calves are most likely to turn to concentrate while trying to meet their taste preferences and energy requirements.

**Table 3:** Effect of the age at first forage consumption on solid and total DMI during the calf period (d 1 to 84)

Item	Treatment				SEM	*P* value		
	CON	EHAY	MHAY	LHAY		Trt	Time^1^	Trt × Time
Stater DMI, g/d	952.4	823.5	829.3	916.9	47.2	0.17	< 0.01	0.37
Hay DMI^2^, g/d	-	94.3	102.3	116.3	20.2	0.75	< 0.01	< 0.01
Hay/Total solid^2^, %	-	19.1	18.0	14.2	1.90	0.19	< 0.01	< 0.01
Total solid feed DMI, g/d	952.9	917.5	942.6	1,033.5	49.5	0.53	< 0.01	0.83
Total DMI, g/d	1,715.0	1,674.6	1,704.8	1,795.3	47.7	0.38	< 0.01	0.69

*DMI* Dry matter intake, *BW* Body weight, *ADG* Average daily gain, *SEM* Standard error mean, *Trt* Treatment

CON = Calves were fed starter concentrate without forage from d 1 to 84, HAY = Starter concentrate and forage were fed to calves free choice from d 1 to 84; EHAY = Calves began to consume forage at the earliest (from d 4 to 6), MHAY = Calves began to consume forage from d 7 to 9, LHAY = Calves began to consume forage at latest (from d 10 to 15) time possible

^1^For all variables, data were summarized by week

^2^Since CON were not fed hay, data analysis was restricted to the HAY groups only

The least square means for nutrient intakes are shown in Table 4. The treatment had no effect on CP, starch, fat and ME intakes during the overall experimental period. However, a significant treatment effect for NDF (*P* < 0.01) was found, and a treatment and time interaction was significant for NDF, peNDF and ADF intake (*P* < 0.01), respectively. Calves fed LHAY had greater NDF intake than CON, but similar intakes to EHAY and MHAY groups during calf period. Specifically, no significant differences were found in NDF intake among treatments before 7 weeks of age, while from week 8 of age, a higher NDF intake was found in LHAY calves than CON calves. From week 11, calves in all HAY groups had a higher NDF intake than CON calves.

**Table 4:** Effect of the age at first forage consumption on nutrient intake during the calf period

Item	Treatment				SEM	*P* value		
	CON	EHAY	MHAY	LHAY		Trt	Time^1^	Trt × Time
Calf period (d 1 to 84)								
CP intake, g/d	426.0	401.2	406.8	425.8	10.2	0.19	< 0.01	0.44
Fat intake, g/d	251.0	248.3	250.6	254.8	2.20	0.26	< 0.01	0.23
Starch intake, g/d	307.7	266.6	271.7	293.6	15.3	0.18	< 0.01	0.40
NDF intake, g/d	162.1^a^	184.5^ab^	191.7^ab^	209.6^b^	11.2	0.05	< 0.01	< 0.01
peNDF intake^2^, g/d	-	17.5	19.0	21.6	3.27	0.76	< 0.01	< 0.01
ADF intake, g/d	100.1	110.5	114.4	125.0	6.50	0.09	< 0.01	< 0.01
ME intake, Mcal/d	6.88	6.68	6.78	7.06	0.13	0.24	< 0.01	0.53

CON = Calves were fed starter concentrate without forage from d 1 to 84, HAY = Starter concentrate and forage were fed to calves free choice from d 1 to 84; EHAY = Calves began to consume forage at the earliest (from d 4 to 6), MHAY = Calves began to consume the forage from d 7 to 9, LHAY = Calves began to consume forage at latest (from d 10 to 15) time possible

^1^For all variables, data were summarized by week

^2^Since CON were not fed hay, data analysis was restricted to the HAY groups only

^a,b^Means within a row with different superscripts are significantly different (*P* ≤ 0.05)

**Fig. 3:**
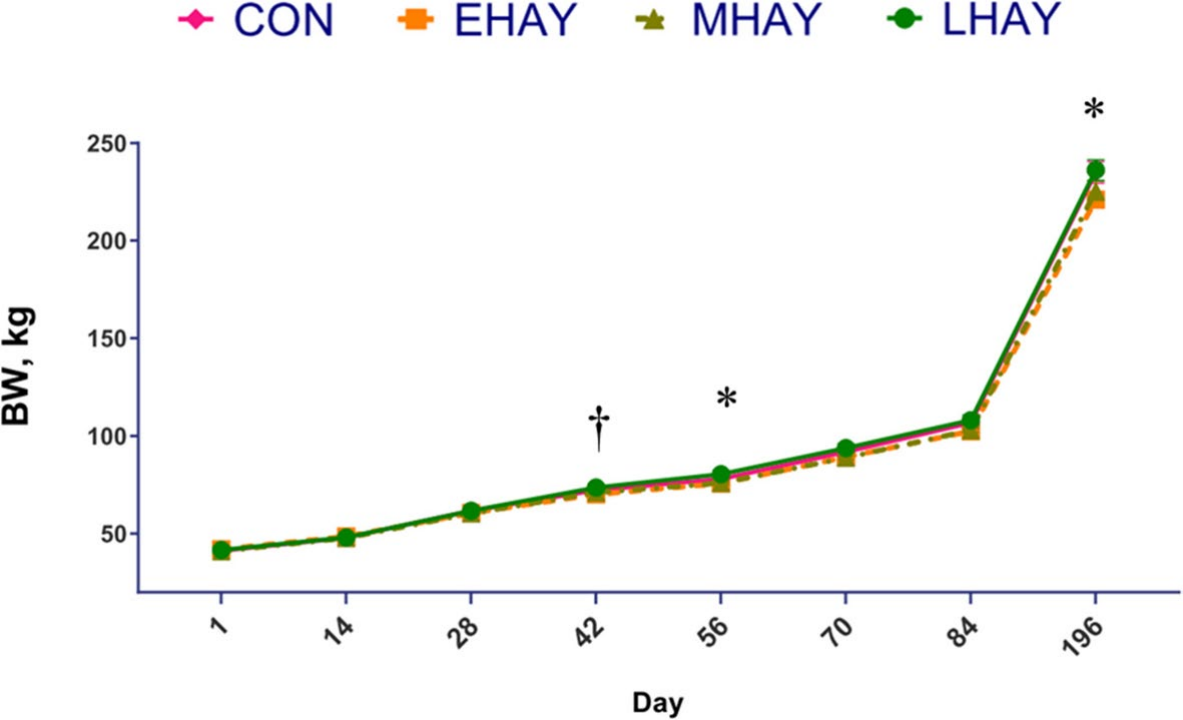
Effect of the age at first forage consumption on BW. Asterisks (*) indicate differences (*P* ≤ 0.05) between groups (d 56: EHAY < LHAY; d 196: EHAY < CON and LHAY). Daggers (†) indicate trends (0.05 ≤ *P* ≤ 0.1) between groups (at d 42: EHAY < LHAY). Error bars represent ± SE. CON = Calves were fed starter concentrate without forage from d 1 to 84, HAY = Starter concentrate and forage were fed to calves as a free choice way from d 1 to 84, EHAY = calves began to consume forage at the earliest (from d 4 to 6), MHAY = calves began to consume the forage from d 7 to 9, LHAY = calves began to consume forage at latest (from d 10 to 15)

The data on growth performance are shown in Table 5 and Fig. 3. A tendency towards significant differences among treatments were found in BW (*P* = 0.06) and ADG (*P* = 0.07), such that, the LHAY and CON calves tended to have a higher BW and ADG compared to EHAY calves (Table 5). Besides, a treatment and time interaction was found for BW (*P* < 0.01), with the LHAY (75.4 kg) calves tending to have higher BW than EHAY calves (69.1 kg, *P* = 0.10) at d 42, LHAY (80.3 kg) having significantly greater weaning BW than EHAY (75.2 kg) at d 56 and EHAY (220.9 kg) having significantly lower final BW compared to CON (235.5 kg) and LHAY (235.8 kg) at d 196.

**Table 5:** Effect of the age at first forage consumption on BW, ADG, FE and structural growth

Item	Treatment				SEM	*P* value		
	CON	EHAY	MHAY	LHAY		Trt	Time^1^	Trt × Time
Overall (d 1 to 196)								
BW, kg	92.1	88.7	89.1	92.7	1.28	0.06	< 0.01	< 0.01
ADG, kg/d	0.83	0.77	0.78	0.84	0.02	0.07	< 0.01	0.92
Withers height, cm	89.2	87.6	87.9	88.3	0.54	0.16	< 0.01	0.12
Heart girth, cm	98.9	98.5	98.5	100.0	0.49	0.14	< 0.01	0.57
Calf period (d 1 to 84)								
FE	2.76	3.02	3.53	2.63	0.38	0.40	< 0.01	0.49

*BW* Body weight, *ADG* Average Daily Gain, *SEM* Standard error mean, *Trt* Treatment, *FE* Feed efficiency, defined as total DMI/ADG

CON = Calves were fed starter concentrate without forage supplementation from d 1 to 84, HAY = Concentrate starter and forage were fed to calves as a free choice from d 1 to 84; EHAY = Calves began to consume forage at the earliest (from d 4 to 6), MHAY = Calves began to consume the forage from d 7 to 9, LHAY = Calves began to consume forage at the latest (from d 10 to 15) time possible

^1^For all variables, data were summarized every two weeks before d 84, data were also collected at the end of the experiment at d 196 for BW and structural growth

For a long time, controversy has existed on whether preweaned calves should be fed forage. While some of the previous studies reported a decrease, others had either an increase or no differences in calf growth performance when forage was added to their diets [11]. The advantages reported in weight gain at a high level of forage have been ascribed to increased gut fill in some studies [12]. In our study, no significant differences in forage and NDF intake were found in HAY groups. Thus, the tendency towards greater ADG and BW in LHAY and CON than EHAY was probably not because of forage induced gut fill. If that was the case, BW should have been similar in HAY groups, while LHAY could have had a lower BW than the CON calves when the same diet was offered. Rather, at d 196 (6 months), the BW was higher in LHAY than EHAY, despite all calves being fed the same diet in the last 3 months of the experiment. These results suggested that introducing hay a few days later after birth (from week 2) and feeding upto 3 months could result in improved BW later in life (6 months). Maktabi et al. found out that calves fed 10% of hay from 4 days of age had a lower ADG and BW compared to calves fed calf starter [42], probably because the lower digestion rate of forage than concentrate starter reduces the availability of nutrients [43]. Although in the current study, there were no significant differences in feed and nutrient intakes among the treatments, a lower digestion rate and digestible nutrient intake were observed as discussed in a later section.

### Blood variables

Concentrations of energy metabolism-related blood metabolites, such as glucose, BHB and NEFA at d 84 of age, were not different (Table 6), implying that calves were similar in energy status [44]. Similarly, previous studies have reported a lack of differences in glucose concentrations between calves partially fed forage and solely fed calf starters [18, 45, 46]. In contrast, controversy concerning BHB remains; some studies that supplemented forage in the diet of calves had similar blood BHB [24, 46], while others reported a greater blood BHB [47–49]. It is generally believed that with an increase in starter feed intake and rumen development, the concentration of plasma BHB increases as age progresses [50]. Besides, a positive linear relationship between starter feed intake and blood BHB level was observed in calves [51]. Similar blood BHB was consistent with the ruminal butyrate concentration at d 84, suggesting that the metabolic function of the developing rumen wall was comparable between groups and that the calves were equally efficient in converting butyrate to BHB [24, 52].

**Table 6:** Effect of the age at first forage consumption on blood variables related to energy metabolism

Item	Treatment				SEM	*P* value
	CON	EHAY	MHAY	LHAY		Trt
Glucose, mmol/L	6.68	6.60	6.61	6.66	0.11	0.94
BHB, mmol/L	0.42	0.44	0.45	0.44	0.03	0.89
NEFA, mmol/L	0.76	0.80	0.75	0.79	0.04	0.72

*BHB* Beta-hydroxybutyric acid, *NEFA* Nonesterified fatty acids

CON = Calves were fed starter concentrate without forage from d 1 to 84, HAY = Starter concentrate and forage were fed to calves free choice from d 1 to 84, EHAY = Calves began to consume forage at the earliest (from d 4 to 6), MHAY = Calves began to consume the forage from d 7 to 9, LHAY = Calves began to consume the forage at latest (from d 10 to 15) time possible

### Rumination behavior development and rumen fermentation

Table 7 presents the age at which calves started ruminating. The calves in the EHAY group tended to begin ruminating the earliest, while those in CON were the latest (EHAY = 12.3 d; CON = 15.5 d, *P* = 0.06), indicating that the earlier the calves started to consume hay, the earlier the rumination occurs. In line with our results, a recent study reported that the age at first rumination was positively correlated with the age at which calves began to eat bedding straw [29], suggesting that consuming physically effective fiber earlier in life was key to initiating the rumination behavior. Rumination behavior, which distinguishes ruminants from monogastric animals, accompanies the development of the rumen and intake of solid feed in calves [53]. This physiological process promotes the breakdown and decomposition of feed and stimulates digestion in ruminants [30].

**Table 7:** Effect of the age at first forage consumption on daily rumination time and the age at first rumination and starter consumption

Item	Treatment				SEM	*P* value		
	CON	EHAY	MHAY	LHAY		Trt	Time	Trt × Time
Daily rumination time, min/d	281.3	278.8	283.8	279.5	9.11	0.97	< 0.01	0.04
The age at first rumination, d	15.5	12.3	13.9	14.1	0.94	0.06	-	-
The age at first consuming starter, d	6.76	6.04	6.76	6.75	0.49	0.61	-	-
The age at first consuming forage, d	-	5.11^a^	7.91^b^	12.1^c^	0.22	< 0.01	-	-

CON = Calves were fed starter concentrate without forage from d 1 to 84, HAY = Starter concentrate and forage were fed to calves free choice from d 1 to 84, EHAY = Calves began to consume forage at the earliest (from d 4 to 6), MHAY = Calves began to consume the forage from d 7 to 9, LHAY = Calves began to consume the forage at latest (from d 10 to 15) time possible

^a–c^Means within a row with different superscripts are significantly different (*P* ≤ 0.05)

**Fig. 4:**
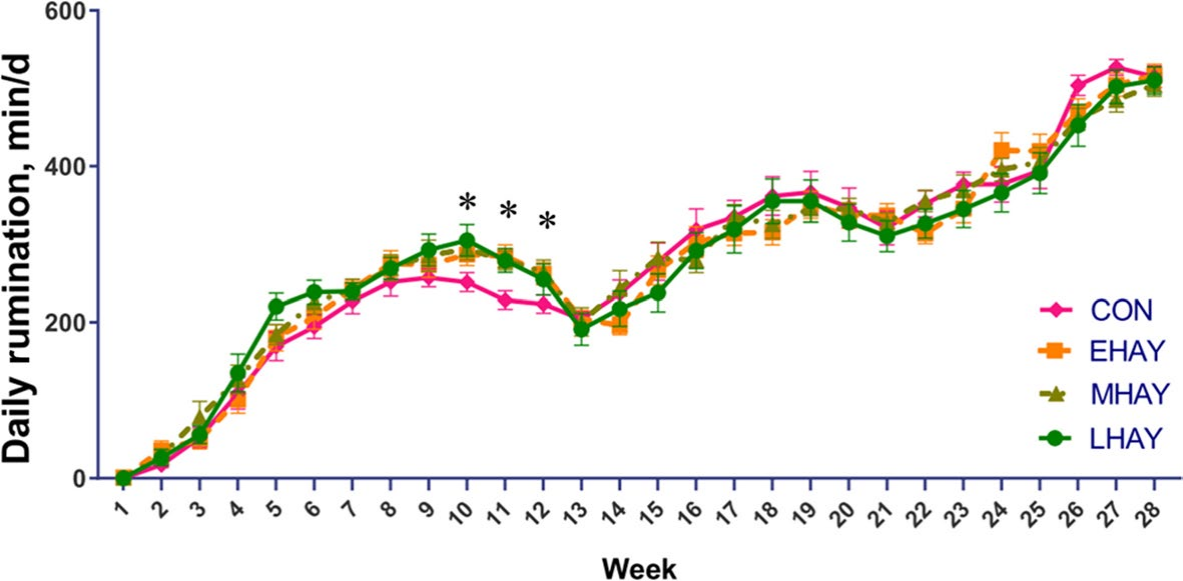
Effect of age at first forage consumption on daily rumination time during the calf, top-dressing and TMR feeding periods. Asterisks (*) indicate differences (*P* ≤ 0.05) between groups (weeks 10: CON < LHAY; weeks 11 and 12: CON < EHAY, MHAY and LHAY). Error bars represent ± SE. CON = Calves were fed starter concentrate without forage from d 1 to 84, HAY = Starter concentrate and forage were fed to calves as a free choice way from d 1 to 84, EHAY = calves began to consume forage at the earliest (from d 4 to 6), MHAY = calves began to consume the forage from d 7 to 9, LHAY = calves began to consume forage at latest (from d 10 to 15). Calves were housed in individual hutches from d 1 to 84 (weeks 1 to 12) and then transferred to a barn and fed calf starter top-dressed with hay (TDR, from d 85 to 133, week 13 to 19) and total mixed ration later (TMR, from d 134 to 196, week 20 to 28)

Despite beginning to consume hay earlier, overall, the EHAY calves did not report improved rumination time compared LHAY, MHAY or CON calves. However, it was worth noting that an effect of treatment and time interaction was found for daily rumination time (*P* = 0.04; Table 7, Fig. 4). Rumination time was similar among treatments, up until calves were completely weaned off milk. After complete milk withdrawal, the CON calves spent less time ruminating in weeks 10, 11 and 12 compared with the calves fed hay. Afterwards, when all calves were transferred to top-dressing (week 13) or TMR diets (week 20), the effect of treatment on rumination behavior faded away, with the rumination time in the CON calves catching up with the other treatments. We also observed a dramatic drop in rumination behavior in all calves at weeks 13 and 20 (Fig. 4). In both young calves and mature dairy cows, rumination is a good indicator of the physiological status of the animal. Rumination time is a relevant marker in evaluating health, rumen development and welfare in calves [54], and a decrease in rumination time has been associated with stress, anxiety and various diseases in mature cows [55]. Thus, a sudden decrease in rumination time in weeks 13 and 20 was probably due to stress arising from the dietary and housing changes, as calves were moved to a new barn in week 13, while the diet was changed at both time points. In the current study, we recorded the rumination time using acceleration sensors instead of traditional visual observations. With the continuous progress in science and technology, breakthroughs are emerging in how rumination behavior is recorded and interpreted in young and mature animals. An automated rumination monitoring system can decrease labor costs and provide convenience for long-term observations. Previous work has evaluated the feasibility of using acceleration sensors to monitor the rumination behavior in calves [35–37]. These studies showed that the sensors were more reliable in identifying and detecting rumination behavior. Future studies should monitor the long-term effects of rumination behavior in early life on growth and health to understand the development of dairy calves better.

The least square means for rumen fermentation parameters are shown in Table 8. No treatment differences were found for rumen pH, NH_3_-N and VFA during the overall period. The CON calves tended to have a lower acetate to propionate ratio than LHAY calves during the overall period (*P* = 0.08). Most studies have reported an increase in acetate to propionate ratio when forage is included in the diet of young calves [13, 56]. Forage is bulkier and has lower digestibility compared to the starter concentrate. Forage consumption might lead to an increase in NDF and effective fiber intakes, which stimulates rumination and chewing activity in calves [57]. Subsequently the flow of saliva into the rumen and rumen buffering are improved [13], which might affect the rumen microbial composition and fermentation environment. A previous study found that dietary forage inclusion increased the abundance of cellulolytic bacteria that produce more acetate and less propionate in the rumen [58]. In contrast to most of the previous studies, we did not observe any differences in the pH of the treatments at the sampled time points (d 1, 7, 35 and 196); only a trend towards higher pH was found in LHAY calves than CON calves at d 84 (data not shown). As calves received a relatively high level of milk allowance from d 1 to 35 (8 or 10 L/d) in the current study, leading to a small amount of hay intake and only a few numerical differences among the treatments (-, 4.13, 3.93 and 7.49 g for CON, EHAY, MHAY and LHAY respectively, data not shown), which might not evoke changes in rumen fermentation [59]. Previously, in calves fed either milk replacer and hay or milk replacer, hay, and a calf starter, no differences were observed in the rumen pH among the treatments [60]. However, in the same study, the authors showed that hay intake, as little as 80 g/d could effectively increase the rumen pH and mitigate ruminal acidosis in calves [60]. Thus, we speculate that lack of differences in rumen pH before weaning could be ascribed to similar and smaller amount of hay intakes in the treatment groups. Upon transitioning to the same diet after d 84, given that rumination plays a key role in regulating the rumen pH, a similar rumination pattern observed in the current study might explain why rumen pH was similar at 6 months of age. These results were consistent with our previous study investigating the effect of early feed exposure on long-term rumen pH, whereby there were no differences between calves offered either concentrate only, hay only or hay plus concentrate in the pre-weaning period even after the TMR diet was introduced to all groups after weaning [40]. Hence, the age at which calves voluntarily consume forage early in life might not affect rumen pH and fermentation in the long term, rather the characteristics of the feed might play a much greater role.

**Table 8:** Effect of the age at first forage consumption on rumen fermentation throughout the experiment (d 1, 7, 35, 84 and 196)

Item	Treatment				SEM	*P* value		
	CON	EHAY	MHAY	LHAY		Trt	Time	Trt × Time
pH	6.16	6.15	6.18	6.18	0.05	0.90	< 0.01	0.49
NH_3_-N, mmol/L	6.41	7.48	6.75	6.88	0.54	0.51	< 0.01	0.65
Total VFA, mmol/L	46.5	45.1	44.3	52.6	5.42	0.76	< 0.01	0.76
Acetate, mol/100 mol	50.8	51.7	50.5	53.0	0.96	0.34	< 0.01	0.51
Propionate, mol/100 mol	29.6	28.0	29.3	26.6	1.02	0.11	< 0.01	0.94
Butyrate, mol/100 mol	14.3	15.2	13.6	16.0	0.92	0.33	< 0.01	0.78
Acetate/Propionate	1.85	2.04	1.93	2.14	0.08	0.08	< 0.01	0.85

*CON* Calves were fed starter concentrate without forage from d 1 to 84, *HAY* Starter concentrate and forage were fed to calves free choice from d 1 to 84, *EHAY* Calves began to consume forage at the earliest (from d 4 to 6), *MHAY* Calves began to consume the forage from d 7 to 9, *LHAY* Calves began to consume forage at latest (from d 10 to 15) time possible

### Total tract digestibility

The results of the effects of age at first forage consumption on apparent nutrient digestibility and digestible nutrient intake are reported in Table 9. Total-tract apparent digestibility of DM, CP and EE did not differ among treatments. However, the digestibility of OM (*P* = 0.03) and starch (*P* = 0.04) were significantly higher in CON and LHAY calves compared with EHAY calves, while MHAY was intermediate. The digestibility of NDF and ADF tended to be higher in LHAY and MHAY calves compared to CON and EHAY. Furthermore, a significantly lower consumption of digestible CP was found in EHAY and MHAY than CON and LHAY calves (*P* = 0.01). On the other hand, CON calves consumed the least amount of digestible NDF and ADF compared to the hay groups which had similar intakes (*P* < 0.01). The consumption of digestible starch was significantly affected by treatment, such that CON and LHAY calves had the highest intakes and EHAY had the least, with MHAY being intermediate (*P* = 0.02). The EE intake was highest in CON calves and least in EHAY with MHAY and LHAY being intermediate (*P* = 0.02).

**Table 9:** Effects of age at first forage consumption on apparent nutrient digestibility and digestible nutrient intake in dairy calves at week 12 of age (*n* = 10 per group)

Item	Treatment				SEM	*P* value
	CON	EHAY	MHAY	LHAY		Trt
Digestibility, %						
DM	76.3	70.3	74.7	76.1	1.89	0.17
OM	79.6^a^	71.9^b^	75.5^ab^	79.5^a^	2.05	0.03
CP	81.0	73.7	74.9	78.8	2.30	0.11
NDF	50.5	45.3	53.8	58.7	3.62	0.08
ADF	55.4	52.1	58.6	64.7	3.35	0.07
Starch	98.7^a^	97.9^b^	98.0^ab^	98.7^a^	0.26	0.04
EE	89.2	82.3	86.4	87.3	1.88	0.17
Digestible nutrient intake, g/d						
DM	2,369.0	2,142.4	2,179.6	2,471.5	86.5	0.14
OM	2,291.2	2,109.2	2,111.1	2,337.8	88.7	0.15
CP	574.3^a^	485.2^b^	492.8^b^	558.6^a^	21.9	0.01
NDF	272.0^a^	301.6^b^	314.6^b^	367.2^b^	17.9	< 0.01
ADF	183.9^a^	202.9^b^	209.4^b^	243.0^b^	10.5	< 0.01
Starch	991.8^a^	865.7^c^	889.1^bc^	963.3^ab^	28.4	0.02
EE	36.67^a^	28.7^b^	31.25^ab^	33.14^ab^	1.89	0.02

*DM* Dry matter, *OM* Organic matter, *EE* Ether extract, *SEM* Standard error mean, *Trt* Treatment

*CON* Calves were fed starter concentrate without forage from d 1 to 84, *EHAY* Calves began to consume forage at the earliest (from d 4 to 6), *MHAY* Calves began to consume the forage from d 7 to 9, *LHAY* Calves began to consume forage at latest (from d 10 to 15) time possible

^a–c^Means within a row with different superscripts are significantly different (*P* ≤ 0.05)

The apparent nutrient digestibilities in this trial were in the ranges reported previously in weaned calves [18, 61, 62], while there is a higher ADF digestibility than the NDF digestibility, which is not consistent with previous studies. The probably reason for the high ADF digestibility is because of the calves eat the bedding sawdust, as we did not include the sawdust intake in the digestibility calculation, the ADF digestibility was overestimated. The source of nutrients highly influences the total tract nutrient digestibility early in life. The low fiber starter concentrate has greater amounts of digestible nutrients than the high fiber hay, with the latter diet most likely able to compromise dietary nutrient digestibility [63]. Thus, the significantly lower digestibility of OM and starch in EHAY calves than in CON calves could be attributed to the greater content of NDF in the total solid DM consumed by EHAY calves (20.1%) compared to CON (17.0%, data not shown). Interestingly, although calves in the LHAY group consumed a similar content of NDF in the total solid DM (20.5%) as the EHAY calves, they had a greater total tract nutrient digestibility, similar to the CON calves. This implied that the time of forage consumption might play a much important role in affecting the rumen development and nutrient digestibility instead of the level of forage and NDF consumption. Besides, digestible CP, starch and EE intake were consistently lowest in EHAY calves, further highlighting the negative impact of introducing hay in the first week of life. Such a feeding strategy is most likely to compromise the absorption of nutrients and ultimately the development of the rumen [64] due to the alteration of ruminal microbial profile that is critical in feed digestion [65, 66]. These results could partially explain the tendency towards lower ADG and BW in EHAY calves compared to LHAY and CON in our study.

Apart from various factors such as forage sources, quality, quantity and particle size [11], our experiment showed that the time calves begin to consume forage voluntarily might play an important role in their feed digestion and growth performance. In previous studies, which supplemented oat hay from 14 days of age showed that DM, OM, NDF and CP digestibility were similar to calves that were offered starter concentrate only [62]. The period calves were introduced to forage in the aforementioned study was similar to the time of LHAY calves (10 to 15 days of age) in the current study. Hence, including or consuming forages from 10 to 15 days of age might not significantly affect the capacity of the gut to digest feed and absorb nutrients compared to the calves fed starter concentrate only. Thus, our results implied that feeding concentrate starter, did not compromise calf performance. Adopting such a feeding strategy might help farms reduce costs of production especially those relate to labor and hay costs compared to providing both starter and hay to calves early in life. However, due to welfare issues that have been raised by some researchers [67, 68], it is imperative that more research is carried out on the most appropriate age to introduce hay in calves. Our results, especially on nutrient digestibility, digestible nutrient intake and growth performance imply that feeding hay in addition to concentrate from the second week could confer better results compared to early than that. However, whether better digestion and growth performance would have occurred if calves had consumed their initial forage much later, such as after 15 days of age, remains to be determined. Furthermore, there is need to explore the role time calves begin to voluntarily consume forage plays on rumen and intestinal morphology and histological development to further elucidate the mechanisms affecting nutrient digestibility in young calves.

## Conclusions

To the best of our knowledge, this is the first study to explore the temporal changes in calves offered ad libitum access to forage and its effect on feed intake, nutrient digestibility, rumination, rumen fermentation and growth performance. Generally, starting to voluntarily consume forage earlier in life tended to negatively affect the growth of the calf in the short- and long-term. These calves might have had limited capacity to digest the solid feed and utilize the digestible nutrients compared to calves that began later or consumed starter only. However, similar rumination time and rumen fermentation were found among groups, suggesting that the differences in growth were not related to the development of rumination behavior. More studies are required to decipher the underlying regulatory mechanisms controlling forage feeding from around 2 weeks of age rather than immediately after birth.

## Data Availability

The datasets during and/or analyzed during the current study are available from the corresponding authors upon reasonable request.
